# CHQ- SocioEmo: Identifying Social and Emotional Support Needs in Consumer-Health Questions

**DOI:** 10.1038/s41597-023-02203-1

**Published:** 2023-05-27

**Authors:** Ashwag Alasmari, Luke Kudryashov, Shweta Yadav, Heera Lee, Dina Demner-Fushman

**Affiliations:** 1 King Khalid University, Abha, Saudi Arabia; 2 University of Minnesota, Minneapolis, USA; 3 University of Illinois Chicago, Chicago, USA; 4 University of Maryland, College Park, USA; 5 National Library of Medicine, National Institutes of Health, Bethesda, USA

**Keywords:** Research data, Social sciences

## Abstract

General public, often called consumers, are increasingly seeking health information online. To be satisfactory, answers to health-related questions often have to go beyond informational needs. Automated approaches to consumer health question answering should be able to recognize the need for social and emotional support. Recently, large scale datasets have addressed the issue of medical question answering and highlighted the challenges associated with question classification from the standpoint of informational needs. However, there is a lack of annotated datasets for the non-informational needs. We introduce a new dataset for non-informational support needs, called CHQ-SocioEmo. The Dataset of Consumer Health Questions was collected from a community question answering forum and annotated with basic emotions and social support needs. This is the first publicly available resource for understanding non-informational support needs in consumer health-related questions online. We benchmark the corpus against multiple state-of-the-art classification models to demonstrate the dataset’s effectiveness.

## Background & Summary

Health Consumers may turn to online resources to satisfy not only their informational needs, but also seek emotional or social support. The benefits of social and emotional support have been well documented in the literature^1–4^. For example, when someone with a severe, life-threatening condition interacts with others who have experienced similar medical conditions, they feel more supported^5^. Although consumers may find the information they need about the disease, such as treatments, symptoms, and side effects on authoritative health-related websites, getting emotional support or social networking support has been shown to add value, but can currently be obtained mostly through online interactions with peers. Previous studies have shown that social support is associated with improved well-being and better health outcomes^6–8^. The advent of medical Question Answering (QA) Systems has been considered as a promising solution and an efficient approach for retrieving relevant and precise answers from several health-related information sources^9^. Automated medical QA may support clinical decision making providing necessary information that otherwise is hard to find. Several research efforts tackled the problem of medical QA and highlighted the challenges related to question understanding, answer retrieval, and answer generation from the perspective of informational needs^9–12^. To the best of our knowledge, no studies were previously dedicated to medical QA and social and emotional support needs.

While such consumer health questions (CHQ) have been included in a variety of earlier studies as part of broader and more diverse QA tasks^9,12,13^, we believe that questions containing social and emotional needs have unique characteristics that require analysis and evaluation in a dedicated study. These characteristics include (i) variety of emotional states (e.g., fear, anger, confusion, sadness), (ii) emotion causes that may differ from the topic of the question, (iii) different types of social support needs (e.g., emotional, self-esteem, and network), and (iv) a need for different answers based on the social support needs sought.

**Fig. 1:**
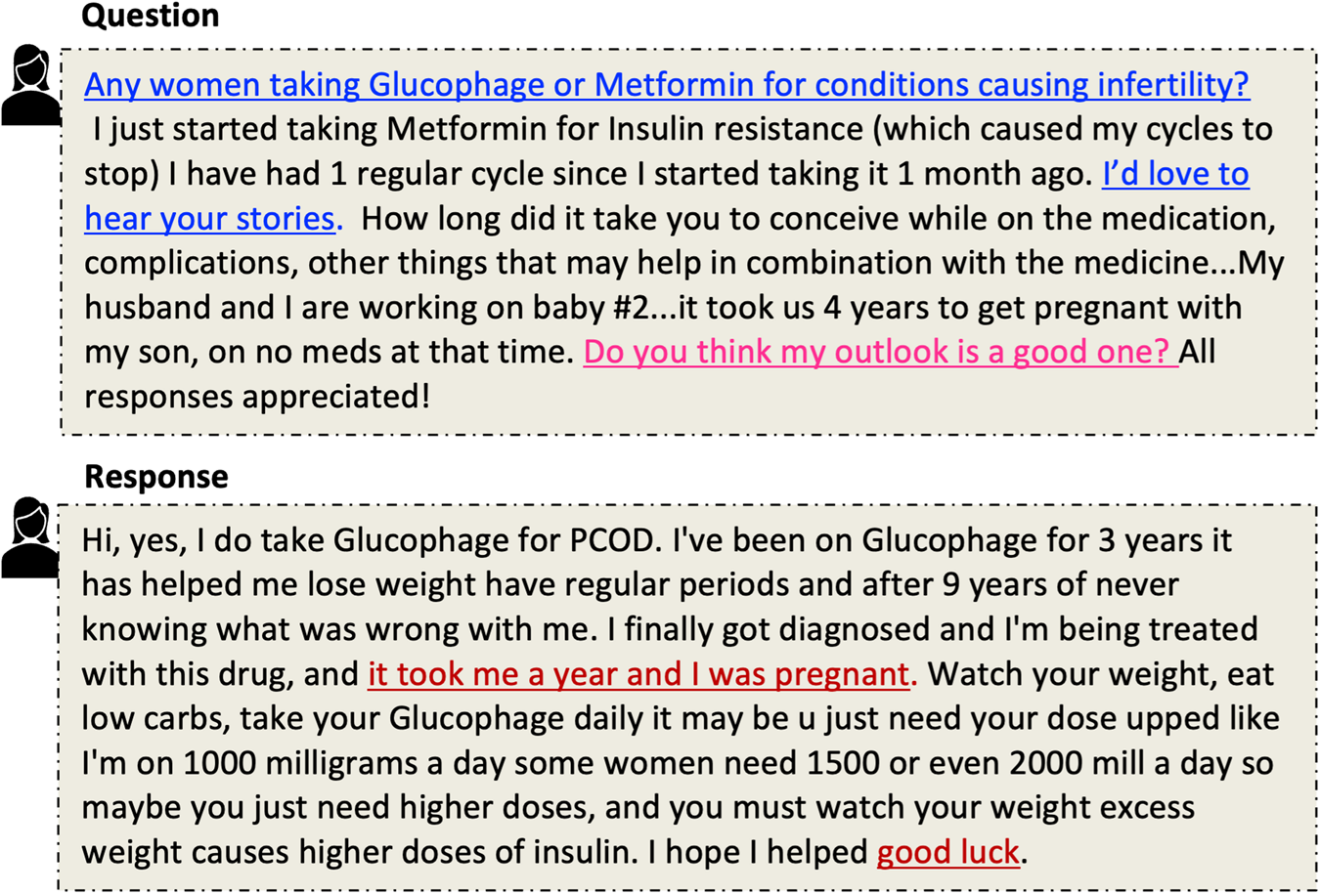
An example from CHQ-SocioEmo dataset including (Question and Response) is posted by Health Consumer while Answer is provided from peer. Different needs in the question are colored differently. NETWORK (colored in blue) and EMOTIONAL (colored in pink) support needs sought in this question. And the NON-INFORMATIONAL(colored in red) support provided in the response.

To illustrate, consider the consumer question from Yahoo! Answers Q&A in Fig. 1. Two different sub-questions can be distinguished: one regarding network support for connecting with women who have taken Glucophage or Metformin and the other seeking emotional support on the outlook for pregnancy. This question includes additional emotions anticipation, shared in the comment on “working on baby #2”, and fear in asking: “Do you think my outlook is a good one?” The response illustrates some level of social and emotional support by providing personal experience and some hope.

Due to the importance of annotated questions for medical QA research, several efforts have focused on this task and collected some relevant datasets^10–12^. To date, only a handful of works have considered emotional and social support in different contexts. For example, Sharma *et al*.^14^ introduced a dataset of 10,000 online conversations in which the responses were annotated using an empathy framework to indicate whether the response post contained no communication, weak communication, or strong communication of empathy. Another dataset introduced by Hosseini and Caragea^15^ includes messages in online support groups annotated as seeking or expressing support. The dataset contains 5,007 sentences, each annotated with one of the three empathy labels: seeking-empathy, providing-empathy, or none. Sun *et al*.^16^ annotated 4,012 answers with a set of mental health support strategies based on psychological counseling theories. Another dataset created by Liu *et al*.^17^ is based on 1,053 statements from a dialog system labeled as help-seeker or supporter mode, based on helping skill theory. These previous studies have provided useful references for emotional and social support corpus development. However, the existing collections’ coverage was insufficient in terms of available annotated consumer health questions to train and build efficient systems to answer beyond informational needs.

**Fig. 2:**
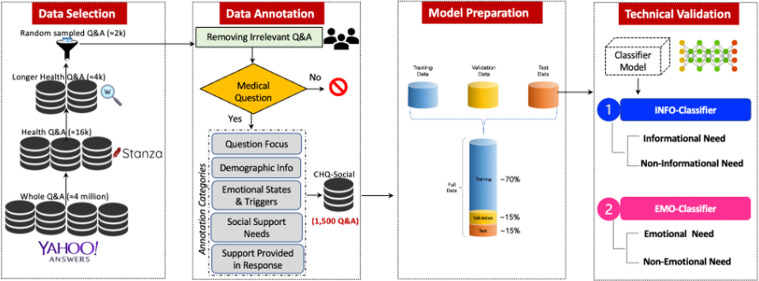
Flow diagram of the *CHQ-*SocioEmo dataset creation and validation.

To this end, we introduce CHQ-SocioEmo, a manually constructed consumer health questions dataset covering social and emotional support needs. CHQ-SocioEmo was collected from a community question answering forum and therefore it is a valuable resource for understanding non-informational support needs in consumer health-related questions online. The CHQ-SocioEmo dataset consists of (a) various aspects of questions annotated based on demographic information, question focus, emotional states and evidence statement, emotion causes, social support needs and (b) annotated answer based emotional support provided. In addition to releasing CHQ-SocioEmo, we include experiments using baseline and state-of-the-art classification approaches, focusing on identification of social support needs, in order to demonstrate the value of CHQ-SocioEmo and benchmark the dataset for future researchers. The flow of building the CHQ-SocioEmo dataset is shown in Fig. 2.

## Methods

### Data collection

We utilized the popular community question answering, “Yahoo! Answers L6” dataset^18^. The dataset is made available by Yahoo! Research Alliance Webscope program to the researchers upon providing consent for using data for non-commercial research purposes only. The Yahoo! Answers L6 dataset contains about 4.4 million anonymized questions across various topics along with the answers. Additionally, the dataset provides various question-specific meta-data information such as best answers, number of answers, question category, question-subcategory, and question language. Since the focus of this study is on consumer health, we restricted ourselves to the questions whose category is “Healthcare” and the language is “English”. To further ensure that the questions are from diverse health topics and are informative, we devised a multi-step filtering strategy. In the first step of filtration, we aim to identify the medical entities in the questions. Towards this, we use Stanza^19^ Biomedical and Clinical model trained on the NCBI-Disease corpus for identifying medical entities. Next, we selected only those question threads with at least one medical entity present in the question. With this process, we obtained 22, 257 question threads from Yahoo! Answers corpus. In the final step, we remove any low-content question threads. Specifically, we retained the questions having more than 400 characters, because longer questions tend to include a variety of needs and background information of health consumers. The final data includes 5,000 question threads.

### Annotation tasks

We used our own annotation interface for all annotation stages. We deployed the interface as a Heroku application with PostgreSQL database. Each annotator received a secure account through which they could annotate and save their progress. We started with smaller batches of 20 questions, and gradually increased the batch size to 100 questions as the annotators became more familiar with the task. The first 20 questions (trial batch) were the same among all annotators, so the annotators worked on the task in parallel. Their annotations were first validated on a trial batch, and they were given feedback to help them correct their mistakes. They were qualified for the main annotation rounds after demonstrating satisfactory performance on the trial batch. In addition, group meetings were conducted to discuss disagreements and document their resolution before the next batches were assigned.

**The following aspects of the questions were annotated:**

**Demographic information** includes the age and sex mentioned in consumer health questions.

**Question Focus** is the named entity that denotes the central theme (topic) of the question. For example, infertility is the focus of the question in Fig. 1.

#### Emotional states, evidence and causes

Given a predefined set of Plutchik-8 basic emotions^20^, annotators label a question with all emotions contained. The annotators were allowed to assign none, one or more emotions to a single consumer health question, for example, a question could be annotated as exhibiting sadness or a combination of sadness and fear. Below are the included emotional states along with their definitions.

- Sadness: Sadness is an emotional pain associated with, or characterized by, feelings of disadvantage, loss, despair, grief, helplessness, disappointment, and sorrow.
- Joy: A feeling of great pleasure and happiness.
- Fear: An unpleasant emotion caused by the belief that someone or something is dangerous, likely to cause pain, or a threat.
- Anger. It is an intense emotional state involving a strong uncomfortable and non-cooperative response to a perceived provocation, hurt or threat.
- Surprise. It is a brief mental and physiological state, a startle response experienced by animals and humans as the result of an unexpected event.
- Disgust. It is an emotional response of rejection or revulsion to something potentially contagious or something considered offensive, distasteful, or unpleasant.
- Trust. Firm belief in the reliability, truth, ability, or strength of someone or something. That does not include mistrust or trust issues.
- Anticipation. Anticipation is an emotion involving pleasure or anxiety in considering or awaiting an expected event.
- Denial. Denial is defined as refusing to accept or believe something.
- Confusion. A feeling that you do not understand something or cannot decide what to do. That includes lack of understanding or communication issues.
- Neutral. If no emotion is indicated.

Alongside, we distinguish between emotion evidence and emotion cause, and we ask annotators to label both accordingly.

- *Emotion evidence* is a part of the text that indicates the presence of an emotion in the health consumer question, so annotators highlight a span of text that indicates the emotion and cues to label the emotion.
- *Emotion cause* is a part of the text expressing the reason for the health consumer to feel the emotion given by the emotion evidence. That can be an event, person, or object that causes the emotion.

For example, the sentence, “Do you think my outlook is a good one?”, shown in Fig. 1 is evidence for Fear emotion, and the cause of Fear is *infertility*. As can be seen in this example, the evidence and the causes are not always found within one sentence. The annotation interface, however, ties them together.

#### Social support needs

According to Cutrona and Suhr’s Social Support Behavior Code^21^, social support exchanged in different settings can be classified as follows:

- Informational support (e.g., seeking detailed information or facts)
- Emotional support (e.g., seeking empathetic, caring, sympathy, encouragement, or prayer support.)
- Esteem support (e.g., seeking to build confidence, validation, compliments, or relief of pain)
- Network support (e.g., seeking belonging, companions or network resources).
- Tangible support (e.g., seeking services)

Examples of the five social support needs are represented in Table 1.

**Table 1:** Examples of Social Support Needs.

Support Need Types	Examples
Informational	*“How long did it take you to conceive while on the medication?”*
	*“How can I wake up?”*
	*“What causes ankle and leg swelling?”*
Emotional	*“Like I said, I’d really love to have my own child, but do you think all of this would be too much for me to handle mentally?”*
	*“Is that reason for worrying?”*
	*“Can anybody offer reassurance backed with a logical explanation as to whether I should be worrying to this extent?”*
Esteem	*“I want to feel normal again I feel like I’m going crazy since my 3 days off of oxy”*
	*“All I hear from everyone is it’s all in your head”*
	*“I got two opinions, one doctor said OK, the other said not ok, I don’t know what to do please help me.”*
Network	*“Is there anyone out there who doesn’t have the intermenstrual bleeding?”*
	*“Does anyone else have zero appetite when stressed or depressed?”*
	*“Do those with Morgellons disease find that they have bipolar or depression before or after getting Morgellons?”*
Tangible	*“I am wondering if there are any practices out there that have led towards a healing or at least to keep the pain at bay.”*
	*“I’ve also lost my job and have no insurance, any support?”*

**The following aspect of the answers was annotated:**

**Emotional support in the answer**. For each answer, annotators had to read the answer and indicate if it is responding to the emotional/esteem/network/tangible support needs by following:

- Yes: if the answer is responding to the emotional, esteem, network, or tangible support needs. The answers were not judged on the completeness or quality with respect to the informational needs. The text span that cued the annotator to the positive response was annotated in the answer.
- No: if the answer is not responding to the emotional, esteem, network, or tangible support needs.
- Not applicable: if questions only seek informational support needs. Thus, no need for the non-informational aspects of the question to be answered.

### Annotator background

The annotation task was completed by 10 annotators (2 male, 7 female, 1 non-binary). As Table 2 shows, the annotators’ ages ranged from 25 to 74 years old and most of them are in the 25–34 and 45–54 brackets. The distribution of ethnicity is 4 White, 3 Asian, 2 Black and 1 Two or more races. In consideration of the diversity, we chose to have annotators from different areas of expertise including biology/genetics, information science/systems, and clinical research. All annotators have a higher educational degree and 60% of them have a doctorate degree. They had a working knowledge of basic emotions and received specific annotation training and guidelines. To measure the annotators’ current state of empathy, State Empathy Scale (SES)^22^ was conducted by 9 annotators. It captured three dimensions in state empathy of annotators including affective, cognitive, and associative empathy. According to the instrument, the affective empathy presents one’s personal affective reactions to others’ experiences or expressions of emotions. Cognitive empathy refers to adopting others’ perspectives by understanding their circumstances whereas associative empathy encompasses the sense of social bonding with another person. According to the results shown in Table 3, the annotators were generally in a state of high empathy reported as the average of 3.31 on a 5-point Likert scale, ranging from 0 (“not at all”) to 4 (“completely”). The annotators showed higher cognitive empathy than affective or associative empathy (M affective = 3.06, cognitive = 3.64, associative = 3.22). This result indicates the annotators were capable of ensuring their emotions did not intervene in annotating others’ emotions, and their perception was based on the context described in the medical questions. Table 4 shows descriptive data including mean, standard deviation, confidence interval for the state empathy scale items

**Table 2:** Demographic information of annotators.

	All annotators	Number of annotators
Gender	Men	2
	Women	7
	Non-binary	1
Age	25–34	3
	35–44	1
	45–54	3
	55–64	2
	65–74	1
Race/Ethnicity	White	4
	Black	3
	Asian	2
	Two/more	1
Education	Bachelor	1
	Master	2
	Doctorate	6
	Professional	1

**Table 3:** State Empathy Scale (SES)^22^ (n = 9).

0 = not at all, 4 = completely	mean	SD
Affective empathy	3.06	0.55
Cognitive empathy	3.64	0.26
Associative empathy	3.22	0.07
Overall	3.31	0.29

**Table 4:** Descriptive Data including Mean, Standard Deviation (SD), Confidence Interval for the State Empathy Scale items.

	Mean	S.D.	95% Confidence Interval	
**Affective empathy**				
1. The asker’s emotions are genuine.	4.00	0.00	4.00	4.00
2. I experienced the same emotions as the asker when reading this health-related question.	2.89	0.60	3.35	2.426
3. I was in a similar emotional state as the seeker when reading this health-related question.	2.00	1.32	3.01	0.98
4. I can feel the seeker’s emotions.	3.33	0.87	3.99	2.66
**Cognitive empathy**				
5. I can see the seeker’s point of view.	3.78	0.44	4.11	3.43
6. I recognize the seeker’s situation.	3.67	0.50	4.05	3.28
7. I can understand what the seeker was going through in this health-related question.	3.56	0.73	4.11	2.99
8. The seeker’s reactions to the symptoms are understandable.	3.56	1.01	4.11	2.99
**Associative empathy**				
9. When reading the health-related question, I was fully absorbed.	3.11	0.78	3.71	2.51
10. I can relate to what the seeker was going through in the health-related question.	3.33	0.87	3.99	2.66
11. I can identify with the situation described in the health-related question.	3.44	0.73	4.00	2.88
12. I can identify with the seeker in the question.	3.00	0.71	3.54	2.45

### Inter-rater agreement

To measure inter-annotator agreement (IAA), we sampled 129 questions from the whole collection annotated by three annotators and asked three additional different annotators to annotate the same questions. IAA is calculated using overall agreement. Table 5 shows the overall agreement for emotional states and support needs in the CHQ-SocioEmo dataset. We first looked at the per-emotion IAA and found that sadness, fear, confusion, and anticipation had the lowest inter-annotator agreement, with overall agreement less than 75%. Joy, trust, surprise, disgust, and denial elicited a higher level of agreement, with overall agreement 75% or higher. We also looked at agreement for each category of the social support needs and found that, all categories had substantial agreement, but for the emotional support that had lower overall agreement (57.36%). This is an open-ended task, and the perception is defined by the disparate backgrounds and emotional make-up, therefore we expected moderate agreement as in the other open-ended tasks, such as MEDLINE indexing^23^.

**Table 5:** Overall agreement for emotional states and support needs in the CHQ-SocioEmo dataset.

Emotional States	Overall agreement (%)
Sadness	60.5
Fear	69.8
Confusion	71.3
Denial	75.2
Anger	86.8
Joy	95.3
Disgust	82.2
Trust	92.2
Surprise	87.6
Anticipation	71.3
Neural	89.1
***Support Needs***	
Informational Support Needs	76.74
Emotional Support Needs	57.36
Esteem Support Needs	78.29
Tangible Support Needs	98.44
Network Support Needs	72.86

## Dataset Characteristics

The dataset contains a total of 1,500 questions and answers pairs. The questions include 8.8 sentences per health questions, 156.33 words per question, on average, whereas the answers on average include 7.3 sentences per answer and 156.33 words per answer.

Distribution of the social support needs is imbalanced with 995 questions seeking informational support, 624 questions seeking emotional support, 207 questions seeking esteem support, 444 questions seeking network support, and 15 questions seeking tangible support. Therefore, we group questions of CHQ-SocioEmo dataset into the three dominant support needs including informational needs, emotional needs, and social needs. Because question counts of tangible support needs are very few compared to other needs, we do not include them in the model. Emotional and esteem support needs were used interchangeably in the literature which are having similar definitions and characteristics. Thus, we group the two types of social support needs, emotional and esteem under the same label.

### Biomedical concepts in the questions

UMLS concepts (and associated semantic types) were extracted from the annotated data using Python (3.10.2), MetaMapLite (3.6.2rc6), and the Python MetaMap wrapper. Concepts were filtered out based on semantic type – only concepts that had at least one relevant semantic type were preserved. We used all the semantic types in UMLS (e.g., Disease or Syndrome, Body Location or Region, Symptoms, Intellectual Product, Conceptual Entity, Idea or Concept), the details are available in GitHub. No additional cleaning was done on the extracted concepts. R (4.0.2) and RStudio (2022.02.3 + 492) were used for the creation of visualizations.

**Fig. 3:**
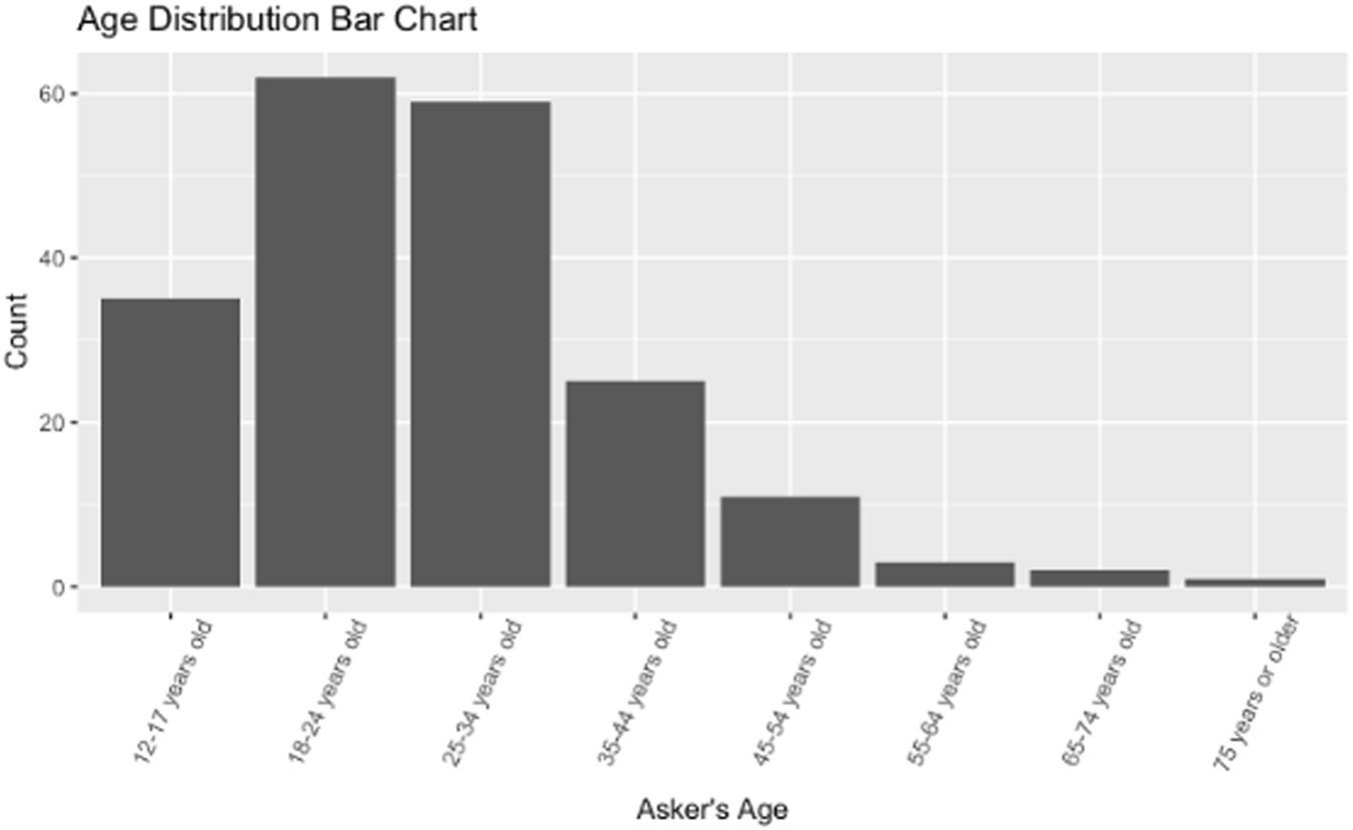
Distribution of age in the CHQ-SocioEmo dataset.

Figure 3 shows the distribution of age ranges in the 198 questions for which the question asker’s age could be determined. 1302 questions did not include information about age and so were excluded from this chart. In the questions for which the asker’s age was known, most of the askers were young adults in the “18–24 years old” as the most common category followed closely by “25–34 years old”.

**Fig. 4:**
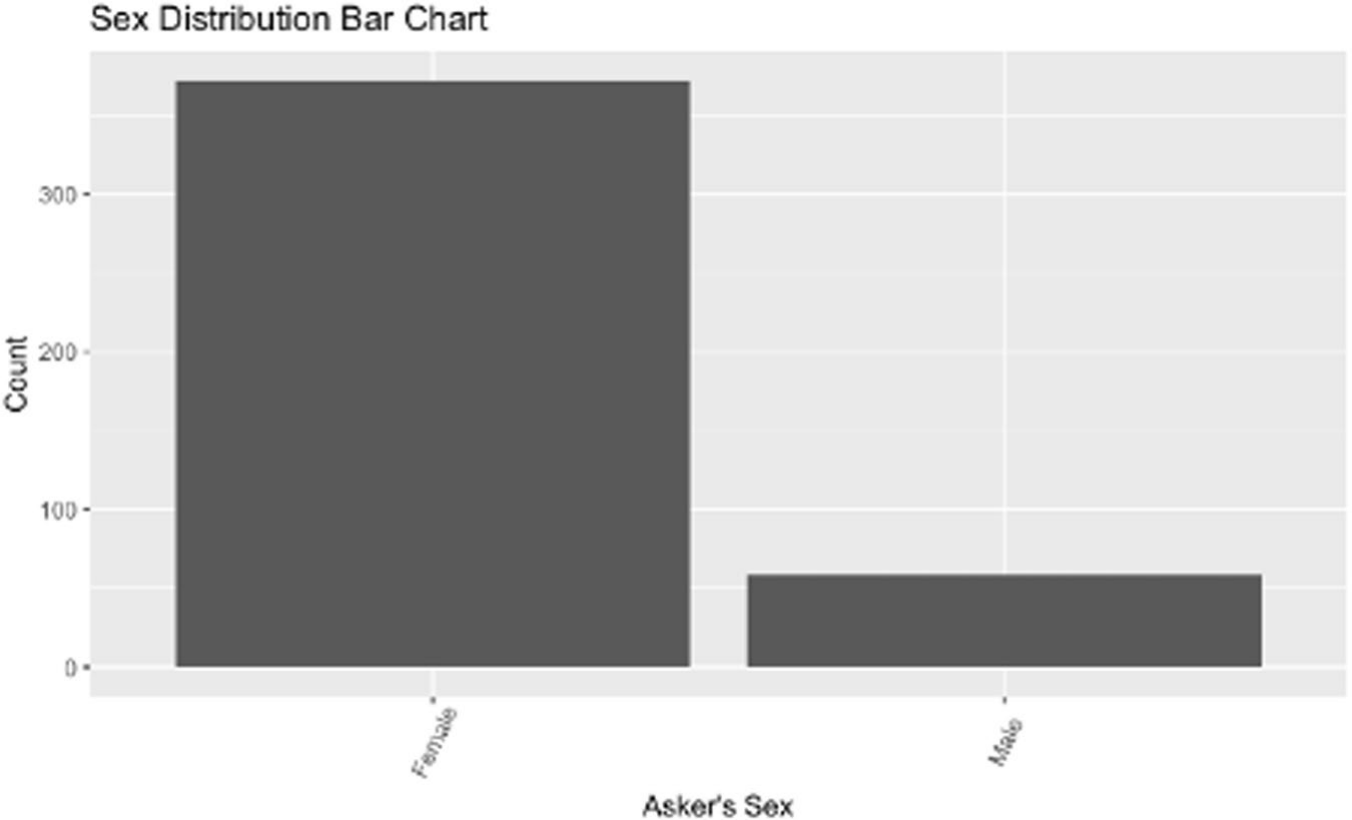
Distribution of sex in the CHQ-SocioEmo dataset.

Figure 4 shows the distribution of sex in the 431 questions for which the question asker’s sex could be determined either through an explicit statement or an implicit discussion of sex-specific health topics. 1069 questions did not include information about the question asker’s sex and so were excluded from this chart. In the questions for which the asker’s sex was known, most askers were identified as female. However, this may be due to a large number of questions about topics related to menstruation and pregnancy and may not accurately reflect the distribution of the full 1500 question sample.

**Fig. 5:**
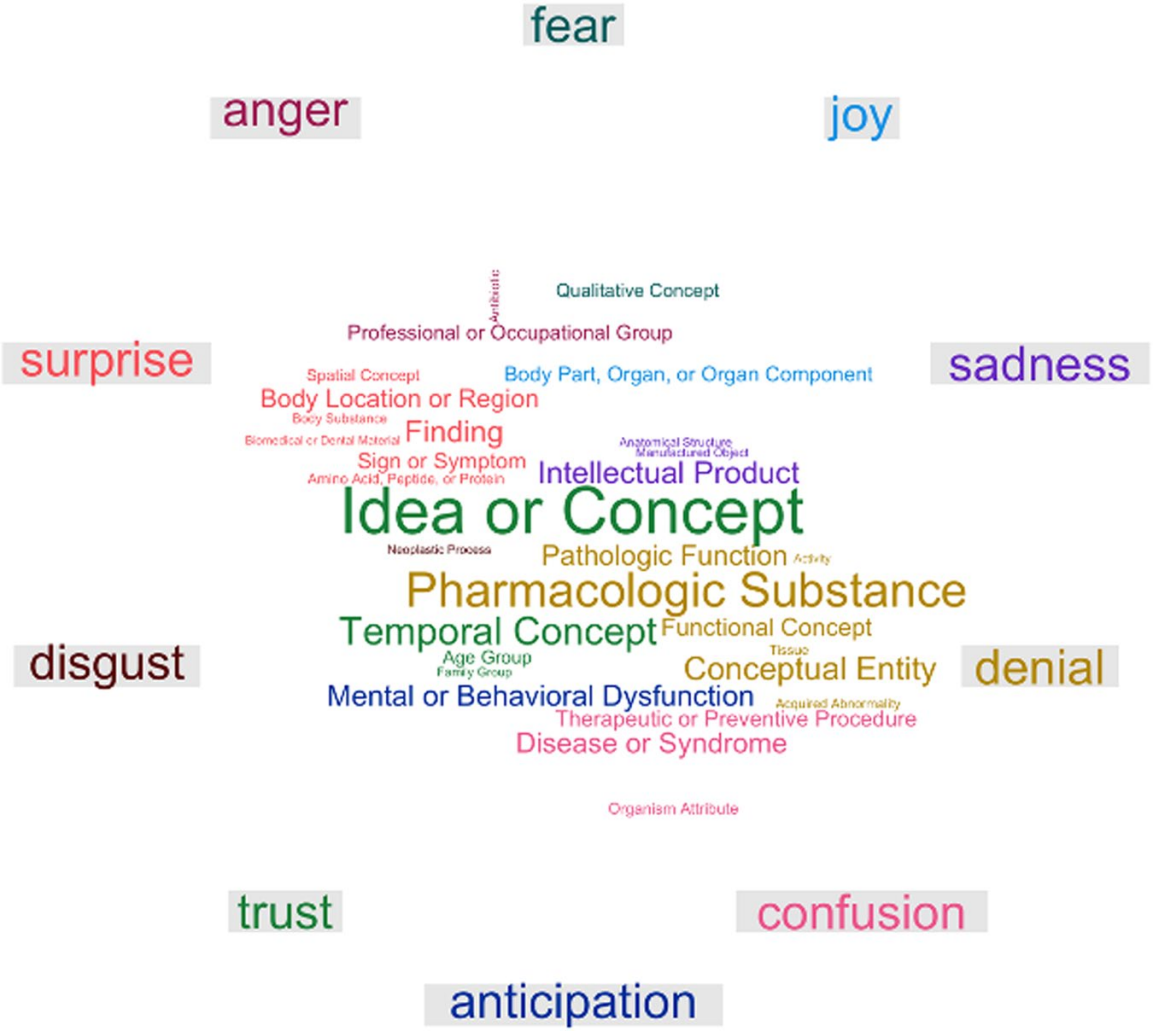
Word clouds of UMLS semantic types associated with emotional triggers. For each basic emotion, the color of the triggers corresponds to the color of the emotion, e.g., Body Location or region and surprise are colored red.

Figure 5 shows a comparison word cloud of UMLS semantic types using emotion causes as the comparison variable. The figure shows that concepts with semantic types of “Qualitative Concept” were more likely to occur in the annotations of emotion triggers for fear, causes of joy were more likely to be associated with “Body Part, Organ, Organ Component”, sadness with “Intellectual Product”, denial with “Pharmacologic Substance”, “Conceptual Entity”, or “Pathologic Function”, confusion with “Disease or Syndrome” and “Therapeutic or Preventive Procedure”, anticipation with “Mental or Behavioral Dysfunction”, trust with “Idea or Concept”, surprise with “Finding”, “Body Location or Region”, and “Sign or Symptom”, and anger with “Professional or Occupational Group”.

**Fig. 6:**
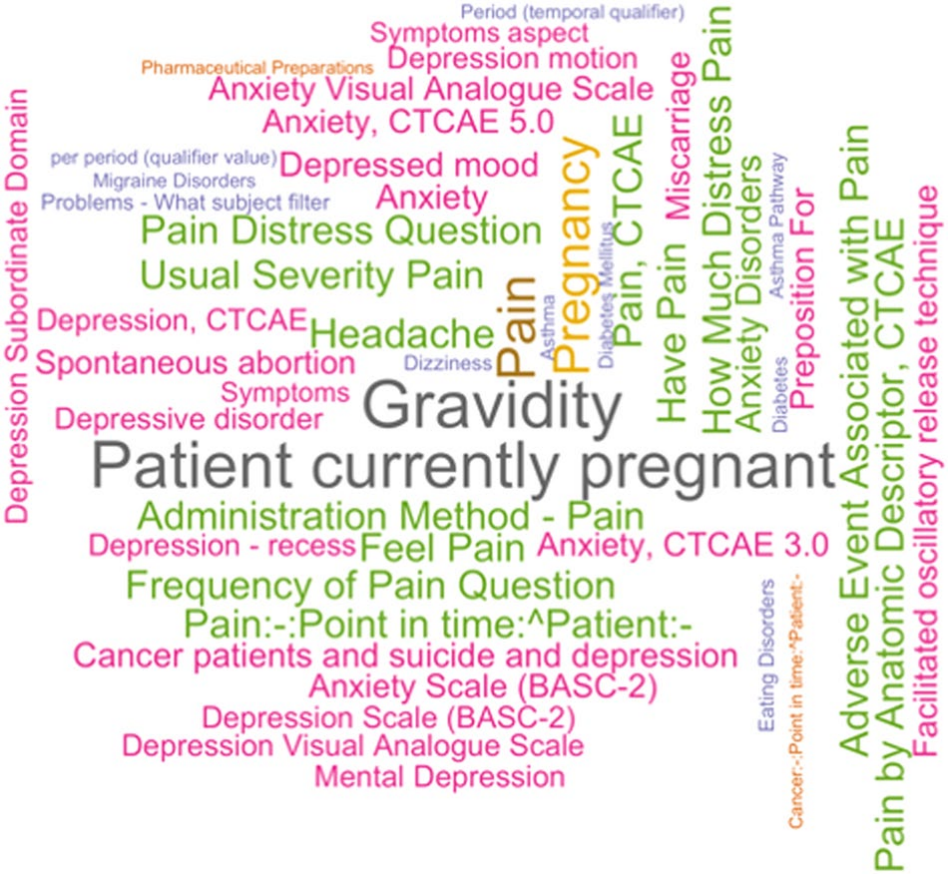
Word clouds of the most frequent UMLS concepts extracted from question focus.

Figure 6 shows a word cloud of the most frequent UMLS concepts extracted from the question focus annotations. It was generated using the word cloud function of the R wordcloud package with larger sizes indicating higher frequency concepts. Concepts with semantic types that were determined to be irrelevant were excluded from this plot, but no additional cleaning was done on the UMLS concepts extracted using MetaMapLite. This figure shows that pregnancy concepts occurred most frequently in the question focus. Various concepts related to pain were also common, as were concepts related to depression, anxiety, asthma, and diabetes.

**Fig. 7:**
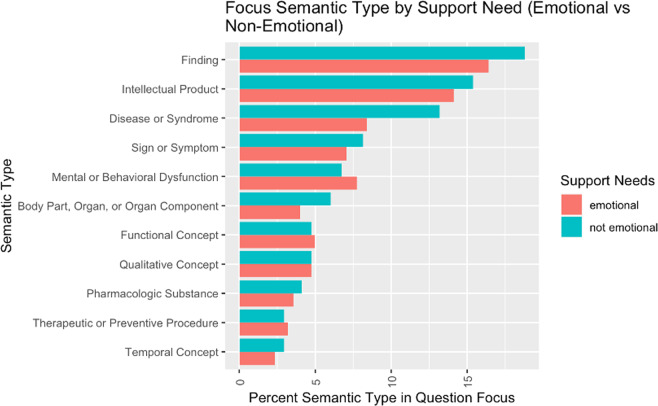
Distribution of the most frequent semantic types of UMLS concepts extracted from question focus.

Figure 7 shows the distribution of the most frequent semantic types of UMLS concepts extracted from the question focus annotations. The chart shows the comparison of distributions among questions that included a request for emotional support needs (“emotional”) and questions that did not include a request for emotional support needs (“not emotional”). The chart uses percentile rather than absolute count measures to allow for comparison among non-equally sized samples. While the distribution is similar for most of the semantic types, questions without emotional support needs were more likely to focus on concepts with the semantic types of “Finding”, “Disease or Syndrome”, and “Body Part, Organ, or Organ Component”, whereas questions with emotional support needs were more likely to focus on concepts with the semantic types of “Mental or Behavioral Dysfunction”.

## Data Records

The Consumer Health Questions Dataset for Social and Emotional Needs (CHQ-SocioEmo) dataset is available through the Open Science Foundation (OSF) at 10.17605/OSF.IO/3DX2S^24^. The dataset is saved in JSON format and is organized according to a health consumer question ID. Each question ID is associated with a nested JSON object. This nested object includes a list of annotated aspects of the questions and answers, which contains question focus, age, gender, fine-grained labels of emotions, emotion evidence, emotion cause, types of social support needs in questions and emotional support in answers. The details of each annotated aspect are provided in the README file. Table 6 shows statistics of the questions and answers in the CHQ-SocioEmo dataset. Additional information about the data structure can be found in the README file included with the OSF archive.

**Table 6:** Average number and standard deviation of words and sentences per CHQs and answers.

Data Type	Count	Words		Sentences	
		Average	S.D.	Average	S.D.
Questions	1,500	156.3	0.5	8.8	0.9
Answers	1,500	133.8	0.9	7.3	0.6

## Technical Validation

### CHQ-SocioEmo benchmarking

We conducted a series of experiments using various classification approaches to benchmark the CHQ-SocioEmo dataset and demonstrate how it can be used to automatically identify non-informational or emotional support needs from questions. We used the pre-trained language models that have shown state-of-the-art performance on the various classification tasks.

- **Naïve Bayes**. Naive Bayes classifier is a simple classifier that was most commonly used in the field of text classfication^25^.
- **LSTM**. Long Short-Term Memory network (LSTM)^26^ is a type of Recurrent Neural Networks (RNN) capable of capturing long-term dependencies. LSTM cell can be used in the sequence-to-sequence model to remember the previous words in the sequence. LSTM processes one word at a time, calculating the possible values for the next word in the sequence.
- **BERT**. The Bidirectional Encoder Representations from Transformers (BERT)^27^ which uses encoders in a transformer as a sub-structure to pre-train models for NLP tasks. BERT’s execution of these tasks is divided into two phases: pre-training for language comprehension and fine-tuning for a specific task. BERT was trained using 16GB datasets from Books Corpus^28^ and the English Wikipedia. BERT has the advantage of being able to handle contextual information extraction due to its bi-directional capability, so it trains faster than other models.
- **RoBERTa**. The Robustly Optimized BERT pre-training Approach (RoBERTa)^29^ is a BERT variant that aims to optimize BERT by tweaking various key parameters in the early form of BERT and training on more data. The model was trained using 160GB of uncompressed text from five English language data sources: the data sources for the BERT model, the CC-News data, the Stories dataset^30^ and the Open Web data.
- **DistilBERT**. Is a distilled version of BERT^31^, with comparable performance but fewer parameters. DistilBERT employs distillation, a compression technique in which a compact model is trained to mimic the behaviour of a larger model.

To model the social support needs in CHQ-SocioEmo, we train classifiers to assign one of 3 labels: informational needs, emotional needs, and social needs. We then perform a 60/20/20 split to build the train, validation, and test sets.

### Benchmarking metrics

We have evaluated the performance of the classifier using precision, recall and weighted-F1 score^32^. Precision is the fraction of correct (relevant) questions among all extracted questions; and recall is the fraction of successfully extracted questions among all correct (relevant) questions. The harmonic mean of precision and recall is called F1 score. The weighted-F1 score is considered for class imbalance in our corpus which calculates the F1 score for each class independently and when it adds them together uses a weight that depends on the number of correct questions of each class^32^. The following are equations for the benchmarking metrics:

$$Precision=\frac{TP}{TP+FP}$$

$$Recall=\frac{TP}{TP+FN}$$

$$F1=2\ast \frac{Precision\ast Recall}{Precision+Recall}$$

$$Weighted-F1\;Score=F1(class1)\ast W1+F1(class2)\ast W2+F1(class3)\ast W3$$

TP is the true positive count (the number of questions that are correctly extracted), FP is the false positive count (the number of questions that are incorrectly extracted), and FN is the false negative count (the number of correct questions that are not extracted).

### Benchmarking results and discussion

We present the performance of models in Table 7. As we can see from the table, DistillBERT outperforms BERT and RoBERTa in two different classes: informational, which achieves an average F1-score of 0.58 and emotional, which achieves an average F1-score of 0.62. However, DistillBERT lags behind RoBERTa in the social category by a mere 0.02%. All models obtain the highest F1-score on emotional needs (F1-score = 0.57, 0.57, 0.62 respectively) compared to informational or social support needs. BERT model performs significantly worse on social needs, obtaining an average F1-score of 0.43.

**Table 7:** Performance results on CHQ- SocioEmo social support needs detection task for all models.

	Informational			Social			Emotional		
	P	R	F1	P	R	F1	P	R	F1
BERT	0.50	0.45	0.46	0.34	0.58	0.43	0.48	0.69	0.57
RoBERTa	0.43	0.66	0.52	0.56	0.57	0.56	0.48	0.69	0.57
DistillBERT	0.63	0.56	0.58	0.58	0.54	0.54	0.62	0.63	0.62

Detecting emotions and emotional needs in text is considered a complex task due to many reasons. One reason is that emotions are subjective and differ among people. Treating the same health consumer question, people may feel different emotions according to their own experience. All these reasons make it hard to detect the emotions, which may impact the evaluation and result in lower performance compared to other three-way classification tasks. The state-of-the-art results for emotion detection tasks range from 44.4% to 70.9%. Yet, the DistillBERT model achieves an average F1-score of 0.58 which is higher, compared to the state-of-the-art datasets such as the GoEmotions dataset. Goemotion achieves an average F1-score of 0.46.

## Usage Notes

We have detailed instructions on how to process the CHQ-SocioEmo dataset in the README file provided with the dataset. The data will support other NLP tasks of interest, such as emotion detection, emotion causes, emotion span detection, question focus, etc. We are unable to directly share the Yahoo original questions due to copyright issues, but instead we provide questions IDs. These questions, however, are publicly available here^1^, and can be accessed after signing the Yahoo agreements.

## Data Availability

The code used to prepare the CHQ-SocioEmo dataset is provided at https://github.com/Ashwag1/CHQ-SocioEmo-, and the source code for the benchmarked experiments can be found with the dataset.
